# Sulfonamide-Resistant Bacteria and Their Resistance Genes in Soils Fertilized with Manures from Jiangsu Province, Southeastern China

**DOI:** 10.1371/journal.pone.0112626

**Published:** 2014-11-18

**Authors:** Na Wang, Xiaohong Yang, Shaojun Jiao, Jun Zhang, Boping Ye, Shixiang Gao

**Affiliations:** 1 State Key Laboratory of Pollution Control and Resource Reuse, School of the Environment, Nanjing University, Nanjing, 210093, China; 2 Nanjing Institute of Environmental Science, Ministry of Environmental Protection of China, Nanjing, 210042, China; 3 School of Life Science and Technology, China Pharmaceutical University, Nanjing, 210009, China; Catalan Institute for Water Research (ICRA), Spain

## Abstract

Antibiotic-resistant bacteria and genes are recognized as new environmental pollutants that warrant special concern. There were few reports on veterinary antibiotic-resistant bacteria and genes in China. This work systematically analyzed the prevalence and distribution of sulfonamide resistance genes in soils from the environments around poultry and livestock farms in Jiangsu Province, Southeastern China. The results showed that the animal manure application made the spread and abundance of antibiotic resistance genes (ARGs) increasingly in the soil. The frequency of sulfonamide resistance genes was *sul*1 > *sul*2 > *sul*3 in pig-manured soil DNA and *sul*2 > *sul*1 > *sul*3 in chicken-manured soil DNA. Further analysis suggested that the frequency distribution of the *sul* genes in the genomic DNA and plasmids of the SR isolates from manured soil was *sul2* > *sul1* > *sul3* overall (*p*<0.05). The combination of *sul*1 and *sul*2 was the most frequent, and the co-existence of *sul1* and *sul3* was not found either in the genomic DNA or plasmids. The sample type, animal type and sampling time can influence the prevalence and distribution pattern of sulfonamide resistance genes. The present study also indicated that *Bacillus, Pseudomonas* and *Shigella* were the most prevalent *sul*-positive genera in the soil, suggesting a potential human health risk. The above results could be important in the evaluation of antibiotic-resistant bacteria and genes from manure as sources of agricultural soil pollution; the results also demonstrate the necessity and urgency of the regulation and supervision of veterinary antibiotics in China.

## Introduction

In the past few decades, veterinary antibiotics have been widely used in many countries to treat disease and promote animal growth. However, this release together with antibiotic-resistant bacteria (ARB) is a great concern recently [1], primarily because the land application of antibiotic-polluted manure in agricultural practice not only introduced bacteria carrying antibiotic resistance genes (ARGs) into the soil but also had a significant effect on the ARB promotion and selection. In the soil, antibiotics provide a positive selective pressure for these bacteria [2]. The horizontal transfer of ARGs between bacteria is an important factor in resistance dissemination [3]. It is worth noting that some ARB in soil and manure are phylogenetically close to human pathogens, making genetic exchange more likely [3]. Evidence from the last 35 years demonstrates that there was consistent correlation between the use of antibiotic-contaminated manure on farms and the transfer of ARGs in human pathogens, as well as the direct shift of ARB from animals to humans [4]. Therefore, ARGs are recognized as new environmental pollutants, and special concern is warranted due to their potential environmental and human health risks.

The used amount of veterinary medicines in China is more than that of other countries. According to a 2007 survey, the usage of antibiotics in livestock was almost half of the total antibiotics produced in China, which was 210,000 tons [5]. It was approximately 10-fold higher than in the USA and approximately 300-fold higher than in the UK [6]. It would be a good chance to analyze the impact of livestock practices on ARGs in the environment in China, where the animal farm was large-scale and the antibiotics usage was great [7]. However, there are few reports on veterinary ARGs in China.

Sulfonamides are synthetic veterinary antibiotics that are the most widely used veterinary antibiotics in China, the European Union and some developing countries due to their low costs [8], [9]. However, sulfonamides were ranged as “High priority” of veterinary medicines, due to the high potential to reach the environment [10]. Sulfonamide resistance is primarily mediated by the *sul*1, *sul*2 and *sul*3 genes encoding dihydropteroate synthetase (DHPS) with a low affinity for sulfonamides [11]–[13]. A wide range of bacterial species harbor these genes, which are located in transposons and in self-transferable or mobilizable plasmids with a broad host range; these genes manifest multiple antibiotic resistance that is co-selected by sulfonamides [14]–[16].

Numerous recent studies have focused solely on the prevalence of sulfonamide resistance genes in bacterial isolates from manured agricultural soils or on the quantification of the total ARGs from environmental soil media to reflect the resistance reservoir. Few studies have systematically covered the identity of sulfonamide-resistant (SR) bacteria and the distribution patterns of sulfonamide ARGs in the total soil DNA and in sulfonamide-resistant bacteria.

The objectives of this study were (i) to determine the influence of the fertilization with antibiotic-polluted manure on the selection of sulfonamide ARB and ARGs and (ii) to investigate the distribution pattern of the *sul*1, *sul*2 and *sul*3 genes in the total soil DNA and the identified SR bacteria. Furthermore, (iii) the identification of the SR bacteria genera and description of the genotypes in each genus were also conducted to identify resistant opportunistic pathogens that increased the risk of ARGs affecting public health. To the best of our knowledge, this is the first comprehensive study of sulfonamide ARB and ARGs in livestock and poultry farms in China. The present study could be important in the evaluation of the pollution of soils used for agriculture by ARB and ARGs from manure; this study also demonstrates the necessity and urgency for the regulation and supervision of veterinary antibiotics in China.

## Materials and Methods

### Sampling

Soil samples from 10 sites were studied, including four pig farms, four chicken farms, one non-arable agricultural area and one mountain forest. The animal feeding farms of different sizes and scales were selected (detailed information about the sampling sites and the person in charge of sampling are given in Table S1 in File S1). The study was permitted and approved by the Ministry of Environmental Protection, China. The land accessed was not privately owned or protected. No protected species were sampled. There were vegetable cultivation area and grain planting area, which were all fertilized with animal manure, in each animal feeding farm. Therefore, two replicates of 1 kg soil samples for each type in every animal feeding farm were collected from depth of 10 to 15 cm, loaded into sterile glass flasks. The soil samples of the same type in different animal feeding farms were mixed (50 g from each source) to processed within 1 to 2 days after collection. The following description was the name rule of samples: (i) samples from the vegetable region of pig farms collected in the winter, the mixture of which was marked as PVW; (ii) samples from the agricultural region of pig farms collected in winter, the mixture of which was marked as PAW; (iii) samples from the vegetable region of pig farms collected in the summer, the mixture of which was marked as PVS; (iv) samples from the agricultural region of pig farms collected in the summer, the mixture of which was marked as PAS; (v) samples from the vegetable region of chicken farms, the mixture of which was marked as CV; (vi) samples from the agricultural region of chicken farms, the mixture of which was marked as CA; (vii) non-arable soils (marked as NA) where manure was not used for a few years near a Nanjing chicken farm; and (viii) forest soil collected from the Fangshan mountain in the Jiangning district of Nanjing (manure and/or antibiotics were not used), which was marked as F. Soil P represents the mixture of soil samples from a pig farm in winter, and soil C is the mixture of soil samples from a chicken farm. The manure (M) was obtained from chickens that were treated with sulfonamides.

For each sample, 100 g was taken for the isolation of SR bacteria and the measurement of sulfonamide residues, and the remainder was stored at 4°C for DNA extraction. Meanwhile, the concentration of sulfonamides in the samples was analyzed in this study using a previously published method [17].

### Viable plate counts

The isolation of SR bacteria from the soil or manure was performed by cultivating bacteria on nutrient broth agar plates containing 60 µg/ml sulfadiazine (SDZ) [15] followed by the spread plate technique [17]. Total bacteria from samples M, F, NA, P and C were cultivated on nutrient broth agar plates without SDZ. In brief, 1.0 ml of each soil sample solution, which was prepared by dissolving 5 g of soil in 45 ml of sterile physiological saline (0.9% NaCl), was mixed with 9 ml of sterile physiological saline. The process was repeated to make additional serial 10-fold dilutions, i.e., 10^−3^, 10^−4^, 10^−5^ and 10^−6^. After 2–5 days of incubation at 37°C, the number of resistant bacteria on the agar plates were counted to calculate the colony-forming units (CFUs) per gram of soil with the following formula: CFU/g soil = 45× average colony number × dilution factor. For subsequent analyses, SR isolates were randomly picked from the plates of each soil sample, with a total of 237 SR bacterial isolates, including 6 isolates from M; 1 isolate from F; 2 isolates from NA; 65, 57, 25 and 25 isolates from PVW, PAW, PVS, and PAS, respectively; and 20 and 36 isolates from CV and CA, respectively. All bacterial strains were stored at −80°C in nutrient broth medium containing 15% glycerol.

### DNA extraction

Total soil DNA was extracted from 0.5 g of soil using a PowerSoil® DNA Isolation Kit (MoBio Laboratories, Carlsbad, California, USA) following the manufacturer’s instructions. SR isolates were cultured at 37°C overnight with constant shaking at 200 rpm/min in 5 ml of LB supplemented with 60 µg/ml SDZ. DNA extraction was performed with 3.0 ml of cultured SR isolates using the TIANamp bacteria DNA kit (Tiangen, Beijing, China). The plasmids were extracted with the Biomiga EZgene™ Plasmid Miniprep kit (Biomiga, USA) following the manufacturer’s protocol. The genomic DNA and plasmids were examined by 1% and 1.5% agarose gel electrophoresis, respectively. Moreover, the λDNA and DNA5000 were used as the marker of genomic DNA and pasmid, respectively. Usually, the molecular weight of genomic DNA was greater than that of the plasmid.

### The detection of the *sul*1, *sul*2, and *sul*3 genes in the SR isolates

The prevalence of the *sul*1, *sul*2, and *sul*3 genes in the genomic DNA and plasmids of the isolates was examined via PCR with gene-specific primers (Table S2 in File S1). The amplification conditions for the *sul*1 and *sul*2 genes were as follows: 94°C for 5 min; 30 cycles of 94°C for 30 s, 69°C for 30 s and 72°C for 45 s; and one cycle of 72°C for 7 min. The amplification conditions for the *sul*3 gene were 94°C for 5 min, 30 cycles of 94°C for 30 s, 52°C for 30 s and 72°C for 60 s, and one cycle of 72°C for 7 min. Gel electrophoresis was performed on 1.5% agarose gels. The CA01 (a bacteria from soil CA) plasmid containing the *sul*1 gene was used as the positive control for the detection of the *sul*1 gene; the M01 (bacteria from chicken manure) plasmid containing the *sul*2 and *sul*3 genes was used as the positive control for the detection of the *sul*2 or *sul*3 genes. *E. coli* DH5α cells were used as the negative control. When the PCR product appeared as a single clear band with the same migration profile as the corresponding gene control, the isolate was counted as positive for that gene.

### Quantitative PCR

The relative abundances of the *sul*1, *su*l2, and *sul*3 genes in the soil DNA were determined in triplicate via SYBR Green-based real-time PCR on a CFX96 Touch Real-Time PCR Detection System. The primer sequences are listed in Table S3 in File S1. Each 10-µl reaction mixture contained 5 µl of SYBR Premix (Cwbio, China), 1 µl of 2 µM forward and reverse primer mix, 1 µl of template, and 3 µl of ddH_2_O. The PCR conditions were 95°C for 10 min, followed by 39 cycles of 95°C for 15 s and 60°C for 60 s. The samples were assessed via 2^−ΔΔCt^ relative quantitative analysis to compare the relative abundance of the *sul* genes among samples. All samples were analyzed in triplicate. The CA01 (a bacteria from soil CA) plasmid containing the *sul*1 gene was used as the positive control for the detection of the *sul*1 gene; the M01 (bacteria from chicken manure) plasmid containing the *sul*2 and *sul*3 genes was used as the positive control for the detection of the *sul*2 or *sul*3 genes. *E. coli* DH5α cells were used as the negative control.

### 16S rRNA sequencing of SR isolates

The complete 16S rRNA gene was used to identify the genera present in the bacterial isolates. Genomic DNA was used as the template for the PCR amplification of the 16S rRNA gene using the universal bacterial 16S rRNA primers 27F and 1492R (Table S2 in File S1). Each 50-µl reaction mixture consisted of 1 to 4 µl of genomic DNA, Taq plus polymerase buffer containing 1.5 mM MgCl_2_, 0.2 mM each of the 4 deoxynucleoside triphosphates (dNTPs), 1 mM each of the 27F and 1492R primers, and 1 U of Taq plus polymerase (Tiangen). PCR was performed using a Bio-Rad thermal cycler under the following conditions: 94°C for 5 min, followed by 30 cycles of 94°C for 30 s, 58°C for 30 s, and 72°C for 1.5 min, and 1 cycle of 72°C for 10 min. The PCR products were separated via electrophoresis on 1.0% agarose gels. The PCR amplicons were sequenced by Sangon (Shanghai, China). A pair-wise 16S rRNA gene sequence similarity was performed using the EzTaxon server (http://www.eztaxon.org/) [18] and NCBI BLAST (http://blast.ncbi.nlm.nih.gov/blast.cgi). A bacterial genus was considered present when a sample 16S rRNA gene sequence was ≥97% identical to the reference sequence of the bacteria in that genus.

### Statistical analysis

The statistical analysis was performed using SAS 9.1. The group mean levels were analyzed via a one-way Analysis of Variance (ANOVA). Statistical significance was defined as a *p*-value≤0.05. This *p*-value was chosen because the standard error associated with CFU plating and qPCR results are generally approximately 5% of the mean. The mean and standard error (SE) displayed in the figures were generated using the means procedure without transformation.

## Results and Discussion

### Enumeration of the total culturable microbial populations and SR Bacteria in the soil

**Figure 1:**
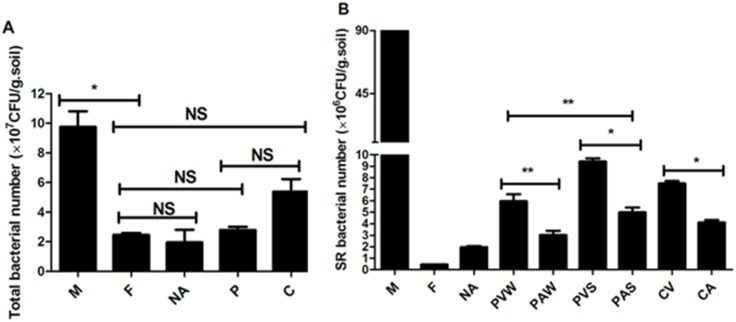
Numbers of cultivable bacteria. (M = Manure, F = Forest, NA = non-arable fied, P = Pig, C = Chicken, W = winter, V = vegetable garden soil, A = agricultural soil; *p≤0.05, **p≤0.01, n = 3; NS, not significant).

The number of total culturable microbial populations on the nutrient agar ranged from 1.96×10^7^ to 9.75×10^7^ CFU/g soil and that of the SR isolates on the nutrient agar ranged from 4.5×10^5^ to 9.0×10^7^ CFU/g soil (Figure 1), which were higher than those of the reported aquaculture-agriculture ponds (3.0×10^4^ to 1.6×10^6^ and 3.0×10^2^ to 4.1×10^4^, respectively) [19]. The higher numbers of total bacteria and SR isolates were found in chicken manure (9.75×10^7^ and 9.00×10^7^, respectively), which was most likely due to the amount of easily accessible nutrients in the manure that stimulated the growth of bacteria [20]. The number of SR bacteria from the soils affected by pig or chicken manure (3.02×10^6^ to 9.40×10^6^ CFU/g soil) was higher than that from non-arable soil (1.96×10^6^ CFU/g soil) or forest soil (4.5×10^5^ CFU/g soil). This difference was most likely due to the application of manure to the soil. Previous studies reported that manure from treated pigs was rich in antibiotics and bacteria carrying ARGs, which were both transferred to the soil via fertilization [3], [10]. Furthermore, the number of SR isolates from the vegetable soils was significantly higher than that from the agricultural soils (5.96×10^6^ and 3.02×10^6^ CFU/g soil for PVW and PAW, respectively; 9.40×10^6^ and 4.98×10^6^ CFU/g soil for PVS and PAS, respectively; 7.50×10^6^ and 4.11×10^6^ CFU/g soil for CV and CA, respectively). Because liquid manure or wastewater was frequently used to irrigate the vegetable region, manure was more frequently applied to the vegetable soils than to the agricultural soils, and the repeated application of manure to the vegetable soils may have increased bacterial resistance. Additionally, the mean number of SR isolates from the winter soils (4.49×10^6^ CFU/g soil for PW) was lower than that from the summer soils (7.19×10^6^ CFU/g soil for PS). This difference most likely occurred because the temperature in the summer is more suitable for the growth of bacteria than that in the winter.

The concentration sums of sulfadiazine, sulfamerazine, sulfathiazole, sulfamethazine, sulfadimethazine and sulfamethoxazole were 4503, 0, 0.536, 35.6, 25.9, 15.8, 12.6, 239 and 193 µg/kg in the mixed samples of M, F, NA, PVW, PAW, PVS, PAS, CV and CA, respectively. The number of cultivable bacteria was not consistent with the concentration of antibiotic sulfonamides in the soil. The pollution level of sulfonamides was found to be significantly higher in chicken farms than in pig farms, but there was no significant difference among the numbers of cultivable bacteria.

### Characterization of SR bacteria

**Figure 2:**
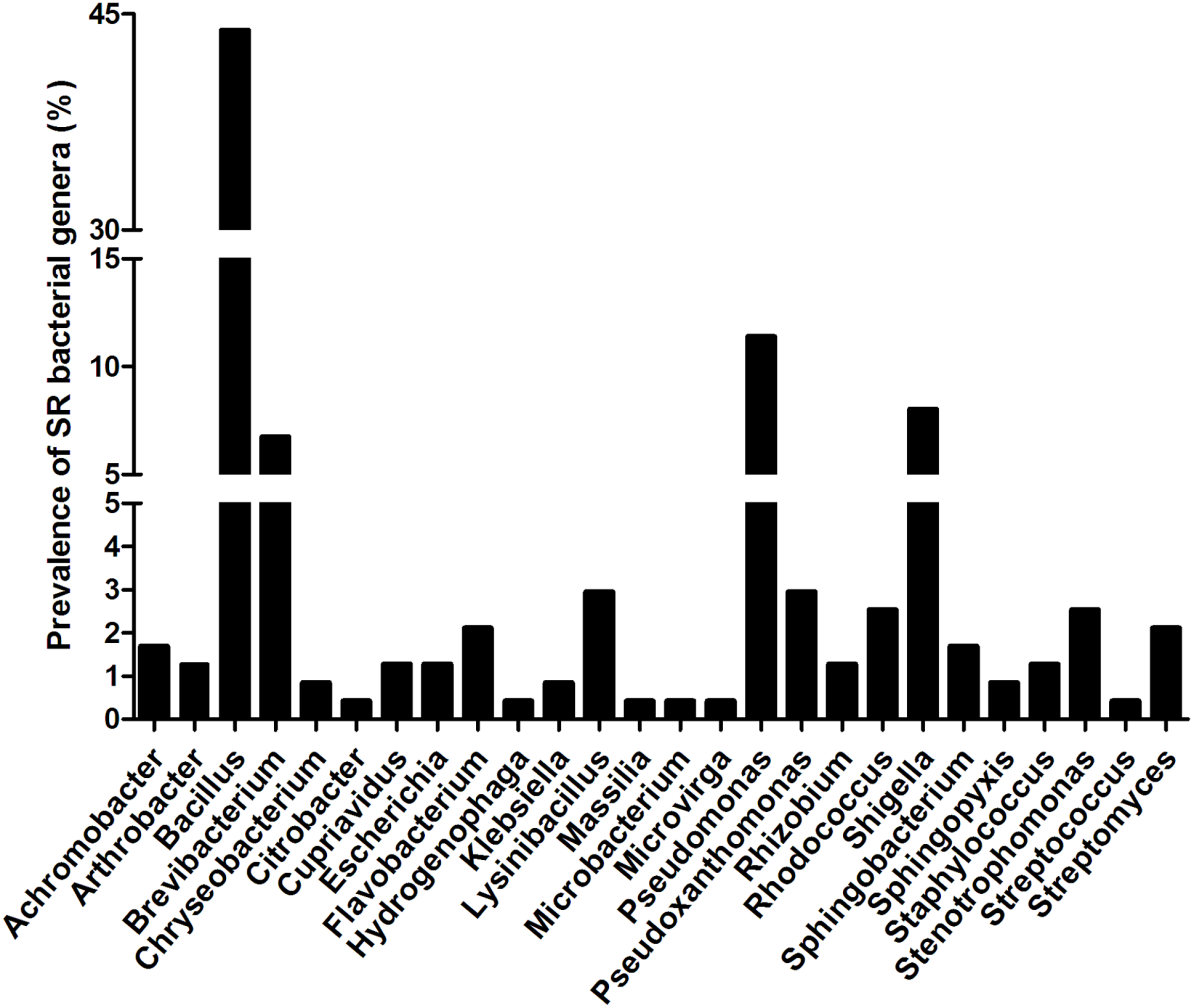
The genera of SR bacteria and their detected frequency in all sampling sites.

All 237 SR isolates that were identified via 16S r RNA belonged to 26 typical soil bacteria genera, including *Achromobacter*, *Arthrobacter*, *Bacillus*, *Brevibacterium*, *Chryseobacterium*, *Citrobacter*, *Cupriavidus*, *Escherichia*, *Flavobacterium*, *Hydrogenophaga*, *Klebsiella*, *Lysinibacillus*, *Massilia*, *Microbacterium*, *Microvirga*, *Pseudomonas*, *Pseudoxanthomonas*, *Rhizobium*, *Rhodococcus*, *Shigella*, *Sphingobacterium*, *Sphingopyxis*, *Staphylococcus*, *Stenotrophomonas*, *Streptococcus*, and *Streptomyces*. *Bacillus* was the most prevalent genus in all 9 environmental samples with a frequency of 43.88%, followed by *Pseudomonas* and *Shigella* (11.39% and 8.02%, respectively; Figure 2). However, it is reported that *Acinetobacter* was abundant in pig wastewater in Vietnam [21]. Both pig- and chicken-manured soil samples were rich in bacteria species; for example, 12 genera were found in PVW and CA (see Figure S1).

### Relative abundance of the *sul* genes in the soils

**Figure 3:**
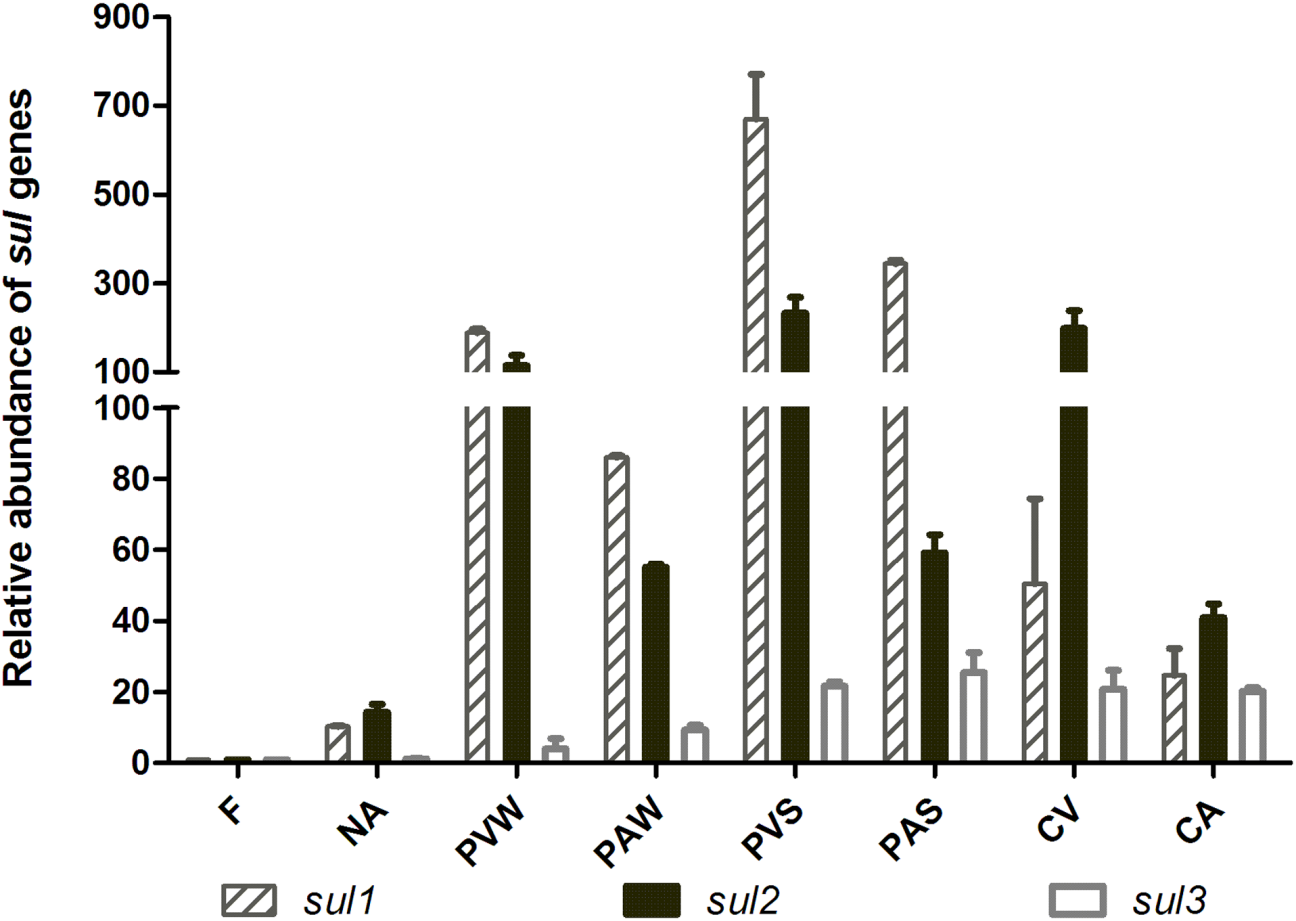
Relative quantity of sulfonamides resistant genes in soils with and without manure treatment.

A qPCR analysis of sulfonamide resistance genes was performed on the total DNA extracted directly from the soil. There was significant variation in the relative quantities of the *sul*1, *sul*2, and *sul*3 genes in the DNA extracted from the eight types of soils (see Figure 3). The DNA from the pig-manured soils (PVW, PAW, PVS and PAS) contained relatively higher copy numbers of *sul*1 than *sul*2. Comparatively, the relative quantity of the *sul*1 gene in the chicken-manured soils was lower than that of the *sul*2 gene. Additionally, the *sul*3 genes were detected at low relative quantities in the DNA extracted from the eight soils but were not detected via PCR in bacteria isolated from forest and pig-manured agricultural (summer) soils. The results of our study were consistent with other reports that demonstrated that the repeated application of manure from pigs or chickens treated with SDZ increased the transfer and abundance of ARGs in the soil [3], [10], [20]. Furthermore, good positive linear correlations were observed between the relative abundance of the *sul2* genes and the number of culturable SR isolates in the soil. For the *sul2* gene and sum of the three *sul* genes, the correlation coefficients (R^2^) were 0.95 and 0.65, respectively (*p*<0.05). However, the abundance of *sul1* and *sul3* showed no significant correlation with the numbers of culturable SR isolates in the soil (R^2^ = 0.44, *p*>0.05 for *sul1* and R^2^ = 0.39, *p*>0.05 for *sul3*). This lack of a correlation could be attributed to the fact that the viable plate counts method only sampled microbes that were culturable and expressed their ARGs under those conditions, so most of the microbes carrying *sul1* and *sul3* genes may not be culturable. The other probable reason was that some “silent” or unexpressed *sul1* and *sul3* genes may be existed in the isolates of soils, which could be horizontally transferred or expressed under other conditions.

In brief, the number of culturable SR isolates in the soil can reflect the total relative abundance of the three *sul* genes, showing that the plate count method was effective in assessing the antibiotic resistance risk of the soil. Therefore, the diversity of ARGs enriched at the farm level should be the focus of more attention.

### Distribution of *sul* genes in SR isolates

The number and percentage of isolates carrying the *sul* genes in their genomic DNA and plasmids are summarized in Table 1 and Table 2. The distribution and spread of SR genes in the soil microbes are sufficiently frequent to warrant special concern. The *sul1*, *sul2*, and *sul3* genes were all detected at a frequency of 100% in the genomic DNA and plasmids of the SR isolates from the manure sample, indicating that ARGs were extensively harbored in the chromosome and mobile genetic elements of the bacteria in manure, leading to the high potential of horizontal gene transfer of ARGs in soil. Interestingly, the *sul2* genes were only present in the genomic DNA of the isolates collected from forest soil and non-arable soil, which had no history of manure application. This finding may be attributed to the notion that the *sul1* and *sul3* genotype in genomic DNA maybe associate with the amended manure. However, the s*ul1*, *sul2* and *sul3* genes were all located in the plasmids of the isolates from non-arable soil but were absent from the plasmids of the isolates from the forest soil; a potential explanation for this difference could be that the bacteria carrying *sul* genes in the manured soil may transfer to the nearby region by aerosolization or runoff, then horizontal transfer occurred in close bacteria via plasmids.

**Table 1:** Distribution of *sul*1, *sul*2 and *sul*3 genes in genomic DNA and plasmid of SR isolates (in samples M, F, NA, CV and CA).

*sul* genecombination		M (n = 6^a^/6^b^)		F (n = 1/0)		NA (n = 2/2)		CV (n = 20/20)		CA (n = 36/36)	
		NO. ofisolates (%)		NO. ofisolates (%)		NO. ofisolates (%)		NO. ofisolates (%)		NO. ofisolates (%)	
		GenomicDNA	PlasmidDNA	GenomicDNA	PlasmidDNA	GenomicDNA	PlasmidDNA	GenomicDNA	PlasmidDNA	Genomic DNA	PlasmidDNA
Singlegenes	*sul*1	0 (0.0)	0 (0.0)	0 (0.0)	0 (0.0)	0 (0.0)	0 (0.0)	0 (0.0)	1 (5.0)	2 (5.6)	0 (0.0)
	*sul*2	0 (0.0)	0 (0.0)	1 (100.0)	0 (0.0)	2 (100.0)	0 (0.0)	2 (10.0)	3 (15.0)	3 (8.3)	2 (5.6)
	*sul*3	0 (0.0)	0 (0.0)	0 (0.0)	0 (0.0)	0 (0.0)	0 (0.0)	0 (0.0)	0 (0.0)	1 (2.8)	0 (0.0)
Twogenes	*sul*1+*sul*2	0 (0.0)	0 (0.0)	0 (0.0)	0 (0.0)	0 (0.0)	0 (0.0)	4 (20.0)	7 (35.0)	10 (27.8)	2 (5.6)
	*sul*1+*sul*3	0 (0.0)	0 (0.0)	0 (0.0)	0 (0.0)	0 (0.0)	0 (0.0)	0 (0.0)	0 (0.0)	0 (0.0)	0 (0.0)
	*sul*2+*sul*3	0 (0.0)	0 (0.0)	0 (0.0)	0 (0.0)	0 (0.0)	1 (50.0)	0 (0.0)	0 (0.0)	1 (2.8)	9 (25.0)
Threegenes	*sul*1+*sul*2+*sul*3										
		6 (100.0)	6 (100.0)	0 (0.0)	0 (0.0)	0 (0.0)	1 (50.0)	12 (60.0)	9 (45.0)	19 (52.8)	23 (63.9)
None		0 (0.0)	0 (0.0)	0 (0.0)	0 (0.0)	0 (0.0)	0 (0.0)	0 (0.0)	0 (0.0)	0 (0.0)	0 (0.0)
Total	*sul*1	6 (100.0)	6 (100.0)	0 (0.0)	0 (0.0)	0 (0.0)	1 (50.0)	16 (80.00	17 (85.0)	27 (75.0)	25 (69.4)
	*sul*2	6 (100.0)	6 (100.0)	1 (100.0)	0 (0.0)	2 (100)	2 (100.0)	19 (95.0)	19 (95.0)	29 (80.6)	36 (100.0)
	*sul*3	6 (100.0)	6 (100.0)	0 (0.0)	0 (0.0)	0 (0.0)	2 (100.0)	13 (65.0)	9 (45.0)	21 (58.3)	32 (88.9)
Total of SRisolate positivefor *sul* genes		6 (100.0)	6 (100.0)	1 (100.0)	0 (0.0)	2 (100.0)	2 (100.0)	19 (95.0)	20 (100.0)	36 (100.0)	36 (100.0)

a = genomic DNA, b = plasmid.

**Table 2:** Distribution of *sul*1, *sul*2 and *sul*3 genes in genomic DNA and plasmid of SR isolates (in samples PVW, PAW, PVS and PAS).

*sul* genecombination		PVW(n = 65/47)		PAW(n = 57/43)		PVS (n = 25/22)		PAS (n = 25/22)	
		NO. ofisolates (%)		NO. ofisolates (%)		NO. ofisolates (%)		NO. ofisolates (%)	
		GenomicDNA	PlasmidDNA	GenomicDNA	PlasmidDNA	GenomicDNA	PlasmidDNA	GenomicDNA	PlasmidDNA
Singlegenes	*sul*1	0 (0.0)	23 (48.9)	0 (0.0)	19 (44.2)	15 (60.0)	0 (0.0)	24 (96.0)	0 (0.0)
	*sul*2	28 (43.1)	3 (6.4)	15 (26.3)	8 (18.6)	1 (4.0)	15 (68.2)	0 (0.0)	16 (72.7)
	*sul*3	0 (0.0)	1 (2.1)	0 (0.0)	2 (4.7)	0 (0.0)	0 (0.0)	0 (0.0)	0 (0.0)
Twogenes	*sul*1+*sul*2	34 (52.3)	11 (23.4)	41 (71.9)	9 (20.9)	1 (4.0)	6 (27.3)	1 (4.0)	6 (27.3)
	*sul*1+*sul*3	0 (0.0)	2 (4.3)	0 (0.0)	0 (0.0)	0 (0.0)	0 (0.0)	0 (0.0)	0 (0.0)
	*sul*2+*sul*3	0 (0.0)	0 (0.0)	1 (1.8)	0 (0.0)	0 (0.0)	1 (4.5)	0 (0.0)	0 (0.0)
Threegenes	*sul*1+*sul*2+*sul*3								
		1 (1.5)	0 (0.0)	0 (0.0)	0 (0.0)	0 (0.0)	0 (0.0)	0 (0.0)	0 (0.0)
None		2 (3.1)	7 (14.9)	0 (0.0)	5 (11.6)	8 (32.0)	0 (0.0)	0 (0.0)	0 (0.0)
Total	*sul*1	35 (53.8)	36 (76.6)	41 (71.9)	24 (55.8)	16 (64.0)	6 (27.3)	25 (100.0)	6 (27.3)
	*sul*2	63 (96.9)	14 (29.8)	57 (100.0)	17 (39.5)	2 (8.0)	21 (95.5)	1 (4.0)	22 (100.0)
	*sul*3	1 (1.5)	3 (6.4)	1 (1.8)	2 (4.7)	0 (0.0)	1 (4.5)	0 (0.0)	0 (0.0)
Total of SRisolate positivefor *sul* genes		63 (96.9)	40 (85.1)	57 (100.0)	38 (88.4)	17 (68.0)	22 (100.0)	25 (100.0)	22 (100.0)

a = genomic DNA, b = plasmid.

For the manured soil, the frequency distribution of the *sul* genes in the genomic DNA and plasmids of the SR isolates investigated overall followed a trend of *sul2* > *sul1* > *sul3* (*p*<0.05). This result was in contrast to several previous studies showing that the *sul1* gene was more prevalent than the *sul2* gene in the DNA from manure and manured soils [10], [15] due to different conditions in various countries. The *sul3* gene was found at low frequencies in our samples, whereas recently, Suzuki et al showed that sul3 was major sul in seawater [22]. Hoa et al. suggested that most of the *sul* genes are located on the chromosome [15]. However, there was no significant difference between the overall percentage of the isolates carrying the *sul* genes located on the genomic DNA and those on the plasmids in our study. It was interesting to note that the frequency order of the *sul1* and *sul2* genes from the isolates of the pig-manured soils for the genomic DNA was opposite that for the plasmids. In the isolates collected from the pig-manured soils in winter, *sul2* was the most prevalent gene located within the genomic DNA (96.9% and 100.0% in PVW and PAW, respectively) followed by *sul1* (53.8% and 71.9% in PVW and PAW, respectively); *sul1* was the most prevalent gene located on plasmids (76.6% and 55.8% in PVW and PAW, respectively) followed by *sul2* (29.8% and 39.5% in PVW and PAW, respectively). However, in the isolates collected from pig-manured soil in summer, the order of *sul1* (64.0% and 100.0% in PVW and PAW, respectively) > *sul2* (8.0% and 4.0% in PVW and PAW, respectively) in the genomic DNA and *sul2* (95.0% and 100.0% in PVW and PAW, respectively) > *sul1* (27.3% and 27.3% in PVW and PAW, respectively) in the plasmids was determined. We concluded that in most isolates, *sul*1 and *sul*2 were located in the different mobile elements and transferred at different rates.

Furthermore, the animal type was a significant factor influencing the expression frequency of *sul* genes, which showed that the frequency in the chicken-manured soil was higher than that in the pig-manured soil (*p*<0.05), which was consistent with the data of the concentration of sulfonamides in the soil.

We also determined the co-presence of any two different *sul* genes on the chromosome and plasmids in a single isolate. The combination of *sul*1 and *sul*2 on the chromosome was the most frequent and was present in PVW, PAW, PVS, PAS, CV and CA (52.3%, 71.9%, 4.0%, 4.0%, 20.0% and 27.8%, respectively), and the *sul*1, *sul*2 and *sul*3 genes were highly co-present on the chromosomes of M, CV and CA (100%, 60.0% and 52.8%, respectively). The co-presence of *sul*2 and *sul*3 was only detected in two isolates from PAW and CA, respectively, and the co-existence of *sul*1 and *sul*3 was not detected in any SR isolates. The *sul*1 and *sul*2 genes were also frequently detected together in the plasmids (23.4%, 20.9%, 27.3%, 27.3%, 35.0% and 5.6% in PVW, PAW, PVS, PAS, CV and CA, respectively). In contrast, the co-presence of *sul*2 and *sul*3 was only detected in NA (50.0%), PVS (4.5%) and CA (25.0%), and the co-presence of *sul*1 and *sul*3 was not found in any plasmids. Furthermore, the three *sul* genes were co-present in the plasmids of M (100%), NA (50.0%), CV (45.0%), and CA (63.9%). We concluded that the combination of *sul*1 and *sul*2 was the most frequent and that the co-existence of *sul*1 and *sul*3 was not found in the genomic DNA or plasmids. Based on these results, the co-presence of the three *sul* genes was only in the isolates from manure and soil from chicken farms, suggesting that there was a positive correlation between the frequency of the co-presence of the three *sul* genes and the time and amount of repeated manure applications.

In summary, the *sul* genes, either individually or in combinations of two or three, were present in the SR isolates at high frequencies. Nearly all plasmids from the SR isolates contained the *sul* genes (with the exception of F). This observation suggests that the resistance that we observed in most cases was linked to plasmids or other mobile genetic elements, which theoretically have transfer potential. The SR isolates could possibly carry these *sul* genes through gene transfer under selection conditions, leading to an increase in antibiotic resistance among bacteria.

### SR bacterial and *sul* genes

The distribution of *sul* genes in bacteria species is listed in Table 3. *Bacillus* was the most prevalent *sul*-positive genus in the soil samples of this study, carrying the *sul* genes in 43.88% of the total isolates; thus, this genus could be the main reservoir of the *sul* genes. This finding was not consistent with other studies that showed that *Acinetobacter* was the dominant genus in aquatic environments (wastewater and shrimp ponds of north Vietnam) and manured agricultural clay soils and slurry samples in the United Kingdom [15], [16]. Except for different environments, what makes the difference of genus may be the different condition of culture, such as 28 or 30°C incubation in these two references, not 37°C. It was reported that *Bacillus* spp. have developed resistance to most antibiotic groups, but only a few species of *Bacillus* have been reported to be sensitive to sulfonamides [23]. *Pseudomonas* and *Shigella* were the second and third most prevalent, carrying the *sul* genes in 11.39% and 8.02% of all isolates, respectively. Ventilator-acquired pneumonia, respiratory tract infections in immunocompromised patients and chronic respiratory infections in cystic fibrosis patients were associated with the *Pseudomonas* species (especially *P. aeruginosa*) [24]. *Enterobacteriaceae* species including *Shigella*, *Klebsiella*, and *Escherichia* have represented some of the most dominant bacterial infections over the last 30 years [24]. In the Henan Province of China, 72.6% of infections were caused by *Shigella* strains in 2006 [25].

**Table 3:** Summary of *sul* genotype of sul-positive bacterial species isolated.

Genus	No. of totalsul-positiveisolates (%)	Source ofisolates	*sul*genotype	No. of sul-positiveisolates
*Achromobacter*	4 (1.69)	NA, PVW, CV	*sul*2	2
			*sul*1 *sul*2	1
			*sul*2 *sul*3	1
*Arthrobacter*	3 (1.27)	CV, CA	*sul*1 *sul*2	1
			*sul*1 *sul*2*sul*3	2
*Bacillus*	104 (43.88)	F, PVW, PAW,PVS, PAS, CV, CA	*sul1*	2
			*sul*2	23
			*sul*1 *sul*2	66
			*sul*2 *sul*3	1
			*sul*1 *sul*2*sul*3	12
*Brevibacterium*	16 (6.75)	PVW, PAW,PVS, PAS	*sul*2	4
			*sul*1 *sul*2	11
			*sul*1 *sul*2*sul*3	1
*Chryseobacterium*	2 (0.84)	PVW, PVS	*sul*1 *sul*2	2
*Citrobacter*	1 (0.42)	CA	*sul*1 *sul*2 *sul*3	1
*Cupriavidus*	3 (1.27)	CA	*sul*1 *sul*2*sul*3	3
*Escherichia*	3 (1.27)	PVW, CA	*sul*1 *sul*2	1
			*sul*1 *sul*2*sul*3	2
*Flavobacterium*	5 (2.11)	CV, CA	*sul*2	1
			*sul*1 *sul*2	1
			*sul*1 *sul*2*sul*3	3
*Hydrogenophaga*	1 (0.42)	PVS	*sul*1 *sul*2	1
*Klebsiella*	2 (0.84)	PAS	*sul*1 *sul*2	2
*Lysinibacillus*	7 (2.95)	PVW, PAW, PAS	*sul*1	1
			*sul*1 *sul*2	4
			*sul*1 *sul*2*sul*3	2
*Massilia*	1 (0.42)	PVW	*sul*2	1
*Microbacterium*	1 (0.42)	PAW	*sul*1 *sul*2	1
*Microvirga*	1 (0.42)	PAS	*sul*1 *sul*2	1
*Pseudomonas*	27 (11.39)	PVW, PAW, CV	*sul*2	1
			*sul*1 *sul*2	23
			*sul*1 *sul*2*sul*3	3
*Pseudoxanthomonas*	7 (2.95)	PVW, PVS	*sul*2	2
			*sul*1 *sul*2	4
			*sul*1 *sul*2*sul*3	1
*Rhizobium*	3 (1.27)	PVS, CV	*sul*1 *sul*2	2
			*sul*1 *sul*2*sul*3	1
*Rhodococcus*	6 (2.53)	PVW, PAW,PVS, PAS	*sul*1 *sul*2	6
*Shigella*	19 (8.02)	CV, CA, M	*sul*1 *sul*2*sul*3	19
*Sphingobacterium*	4 (1.69)	VA	*sul*1 *sul*2	1
			*sul*1 *sul*2*sul*3	3
*Sphingopyxis*	2 (0.84)	PVW, PAW	*sul*2	1
			*sul*1 *sul*2	1
*Staphylococcus*	3 (1.27)	PAW, CA	*sul*1 *sul*2*sul*3	3
*Stenotrophomonas*	6 (2.53)	CV, CA, NA	*sul*2 *sul*3	1
			*sul*1 *sul*2*sul*3	5
*Streptococcus*	1 (0.42)	CV	*sul*1 *sul*2*sul*3	1
*Streptomyces*	5 (2.11)	PVW, PAW, CA	*sul*1 *sul*2	1
			*sul*1 *sul*2*sul*3	4

To the best of our knowledge, this report is the first on *sul* genes in *Chryseobacterium*, *Cupriavidus*, *Flavobacterium*, *Hydrogenophaga*, *Lysinibacillus*, *Massilia*, *Microbacterium*, *Microvirga*, *Pseudoxanthomonas*, *Rhizibium*, *Rhodococcus*, *Sphingopyxis*, *Staphylococcus*, *Streptococcus*, and *Streptomyces* from soils and the first that indicates the widespread presence of ARB in the arable soils of China. Previous studies demonstrated the co-presence of *sul*1, *sul*2 and *sul*3 in a single cell; this was detected in *Acinetobacter*, *Bacillus*, *Psychrobacter*, *Escherichia coli*, and *Salmonella* [15], [16], [26], [27]. In our study, these three *sul* genes were simultaneously found in *Arthrobacter*, *Brevibacterium*, *Citrobacter*, *Cupriavidus*, *Flavobacterium*, *Lysinibacillus*, *Pseudomonas*, *Pseudoxanthomonas*, *Rhizibium*, *Sphingobacterium*, *Staphylococcus*, *Stenotrophomonas*, *Streptococcus*, and *Streptomyces*, with the exception of three genera (*Bacillus*, *Escherichia*, and *Shigella*). This result indicates that the three *sul* genes are common and widely distributed in ARB in soil. Additionally, the *sul*3 gene was detected for the first time in *Achromobacter*, *Chryseobacterium*, *Citrobacter*, *Cupriavidus*, *Flavobacterium*, *Lysinibacillus*, *Pseudoxanthomonas*, *Rhizibium*, *Sphingobacterium*, *Staphylococcus*, *Streptococcus*, and *Streptomyces* from arable soils.

It was revealed that the manured soils could be a reservoir of sulfonamide ARBs and ARGs, according to the observation of high frequency of various combinations of the *sul* genes in bacteria of manured agricultural soils, which may bring potential hazards to human and ecosystem health. Therefore, the diversity of ARGs and ARB enriched at the farm level should be the focus of more attention.

## Conclusion

A comprehensive study of sulfonamide ARB and ARGs in livestock and poultry farms in Jiangsu Province of China revealed that the fertilization with antibiotic-polluted manure had a significant influence on the selection of sulfonamide ARB and ARGs. The sample type, animal type and sampling time may affect the prevalence and distribution rule of SR genes. The results from the identification of the SR bacteria genus and the description of the genotypes in the genus revealed that resistant opportunistic pathogens increased the risk of ARGs affecting public health. Overall, the high frequency of various combinations of the *sul* genes in manured agricultural soil samples of Southeastern China should be the focus of more attention, and the regulation and supervision of veterinary antibiotics are urgently needed in China.

## Supporting Information

Figure S1 Prevalences of SR bacteria belonging to different genera identified in the studied soils.
(TIF)

File S1 Contains the following files: **Table S1**. Detailed information on sampling sites in present study. **Table S2**. Primers for PCR in this Study. **Table S3**. Primers for quantitative PCR in this Study.
(DOC)
