# Supplementary figures and images for: Sulfonamide-Resistant Bacteria and Their Resistance Genes in Soils Fertilized with Manures from Jiangsu Province, Southeastern China
Source: PLoS One. 2014 Nov 18;9(11):e112626. doi: 10.1371/journal.pone.0112626 (PMC4236111; doi:10.1371/journal.pone.0112626)

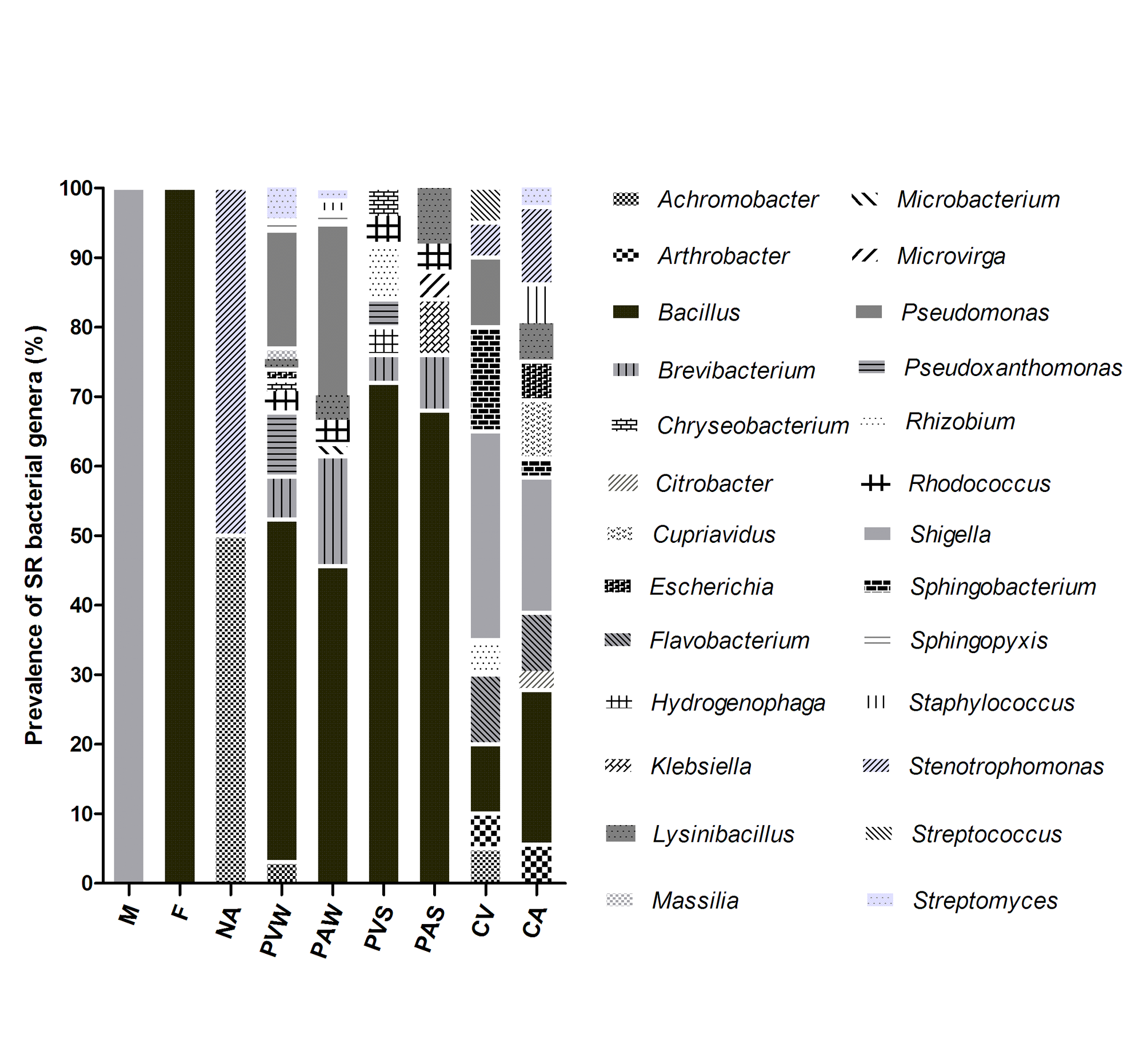

Supplement: Figure S1 — Prevalences of SR bacteria belonging to different genera identified in the studied soils. (TIF) [file pone.0112626.s001.tif]
