# Supplementary material for: Sulfonamide-Resistant Bacteria and Their Resistance Genes in Soils Fertilized with Manures from Jiangsu Province, Southeastern China
Source: PLoS One. 2014 Nov 18;9(11):e112626. doi: 10.1371/journal.pone.0112626 (PMC4236111; doi:10.1371/journal.pone.0112626)
Supplement: File S1 — Contains the following files: Table S1. Detailed information on sampling sites in present study. Table S2. Primers for PCR in this Study. Table S3. Primers for quantitative PCR in this Study. (DOC) [file pone.0112626.s002.doc]

Table S1 Detailed information on sampling sites in present study

| Sample site | geographic(al) position | Farm scale  (pigs/chickens) | Breeding time (years) | Application rates of manure*  (y^-1^) | Application dosage  (ha^-1^) | Agriculture  soil type |
| --- | --- | --- | --- | --- | --- | --- |
| Nanjing pig farm | N32°00′23.0″,  E119°04′01.9″ | 4000 | 7 | 2 | 500-750kg | clay loam |
| Shuyang pig farm | N34°13′42″,  E118°58′54″ | 25000 | 10 | 2 | 15-20m^3^ | clay loam |
| Haian pig farm | N32°37′54.3″,  E120°25′54.1″ | 20000 | 7 | 3 | 15-20m^3^ | clay loam |
| Xuzhou pig farm | N34°22′11.7″,  E118°32′21.8″ | 5000 | 8 | 3-4 | 25-30m^3^ | clay loam |
| Nanjing chicken farm | N32°29'44",  E118°40'05" | 90000 | 12 | 2 | 10-20 m^3^ | clay loam |
| Suqian chicken farm | N33°45'9.2",  E118°14'5.1" | 60000 | 5 | 2 | 10-20 m^3^ | clay loam |
| Haian chicken farm | N32°33'57.2",  E120°35'33.7" | 270000 | 3 | 3 | 10-20 m^3^ | clay loam |
| Xuzhou chicken farm | N34°23'29.5",  E118°25'13.5" | 400000 | 14 | 2-3 | 20-30 m^3^ | clay loam |

*The liquid manure or the wastewater was used for irrigation of vegetable region frequently.

Note: The informations on contacted persons were Mr Wu in Nanjing pig farm (148110641@qq.com), Mr Wang in Shuyang pig farm (wzc1982@126.com), Miss Xu in Haian pig farm ([30893079@qq.com](mailto:30893079@qq.com)), Mr Zhang in Xuzhou pig farm ([zjq989@qq.com](mailto:zjq989@qq.com)), Mr Liu in Nanjing chicken farm ([731696768@qq.com](mailto:731696768@qq.com)), Mr He in Suqian chicken farm ([958320664@qq.com](mailto:958320664@qq.com)), Miss Xu in Haian chicken farm ([1720628848@qq.com](mailto:1720628848@qq.com)) and Mr Zhang in Xuzhou chicken farm (30893079@qq.com).

**Table S2. Primers for PCR in this Study**

| Gene | Primer | Sequence (5′-3′) | Amplicon size (bp) | T_A_  ( ° C) | Reference |
| --- | --- | --- | --- | --- | --- |
| 16s rDNA | 27F | AGAGTTTGATCATGGCTCAG | 1500 | 58 |  |
|  | 1492R | TACGGYTACCTTGTTACGACTT |  |  |  |
| *sul*1 | F | CGGCGTGGGCTACCTGAACG | 433 | 69 |  |
|  | R | GCCGATCGCGTGAAGTTCCG |  |  |  |
| *sul*2 | F | GCGCTCAAGGCAGATGGCATT | 293 | 69 |  |
|  | R | GCGTTTGATACCGGCACCCGT |  |  |  |
| *sul*3 | F | TCAAAGCAAAATGATATGAGC | 787 | 52 |  |
|  | R | TTTCAAGGCATCTGATAAAGAC |  |  |  |
| T_A=_ annealing temperature, F= forward primer, R= reverse primer | | | | | |

**Table S3. Primers for quantitative PCR in this Study**

| Gene | Primer | Sequence (5′-3′) | Amplicon size (bp) | T_A_  ( ° C) | Reference  sequence |
| --- | --- | --- | --- | --- | --- |
| 16s rDNA | qF | CAATGGACGAAAGTCTGACG | 146 | 60 | CU928161 |
|  | qR | ACGTAGTTAGCCGTGGCTTT |  |  |  |
| *sul*1 | qF | TGTCGAACCTTCAAAAGCTG | 113 | 60 | AF071413 |
|  | qR | TGGACCCAGATCCTTTACAG |  |  |  |
| *sul*2 | qF | ATCTGCCAAACTCGTCGTTA | 89 | 60 | M36657 |
|  | qR | CAATGTGATCCATGATGTCG |  |  |  |
| *sul*3 | qF | GGTTGAAGATGGAGCAGATG | 111 | 60 | AJ459418 |
|  | qR | GCCTTAATGACAGGTTTGAGTC |  |  |  |
| T_A=_ annealing temperature, F= forward primer, R= reverse primer, q=quantitative. | | | | | |

**References**

1. Lane D (1991) 16S/23S rRNA sequencing. Nucleic acid techniques in bacterial systematics.

2. Kerrn M, Klemmensen T, Frimodt-Møller N, Espersen F (2002) Susceptibility of Danish Escherichia coli strains isolated from urinary tract infections and bacteraemia, and distribution of sul genes conferring sulphonamide resistance. Journal of antimicrobial chemotherapy 50: 513-516.

3. Heuer H, Smalla K (2007) Manure and sulfadiazine synergistically increased bacterial antibiotic resistance in soil over at least two months. Environmental microbiology 9: 657-666.
